# Roles of Motorcycle Type and Protective Clothing in Motorcycle Crash Injuries

**DOI:** 10.1155/2013/760205

**Published:** 2013-11-17

**Authors:** Mehmet Ozgür Erdogan, Ozgur Sogut, Sahin Colak, Harun Ayhan, Mustafa Ahmet Afacan, Dilay Satilmis

**Affiliations:** ^1^Department of Emergency Medicine, Haydarpasa Numune Training and Research Hospital, 34710 Istanbul, Turkey; ^2^Department of Emergency Medicine, Bezmialem University Hospital, 34098 Istanbul, Turkey

## Abstract

*Background*. The aims of this study were to identify subgroups of motorcyclists with a higher accident risk and evaluate the efficiency of protective clothing for preventing injuries. *Methods*. A 1-year prospective study of motorcycle crashes was conducted beginning in June 2012. Participants were patients involved in motorcycle crashes and admitted to our emergency department. *Results*. A total of 226 patients were included in the study. In total, 174 patients were involved in crashes with light motorcycles. Patients involved in a motorcycle accident without a helmet had a higher incidence of head and maxillofacial trauma. Motorcycle jackets were not protective for systemic injuries (*P* > 0.05) or upper extremity fractures (*P* > 0.05). Motorcycle pants (*P* > 0.05) and motorcycle shoes (*P* > 0.05) were not protective against leg and foot fractures. However, motorcycle protective clothes were protective against soft-tissue injuries (*P* = 0.001). *Conclusion*. Riders of heavy motorcycles rode more safely than riders of light motorcycles. Light motorcycle riders were the most vulnerable and comprised the largest percentage of motorcyclists. Helmets may be effective for preventing head and facial injuries. Other protective clothes were not effective against fractures or systemic injuries.

## 1. Introduction

Motorcycles are the fastest growing sector of motor vehicles worldwide and comprise the majority of all motor vehicles in low- and middle-income countries [1]. Motorcycles are an important part of social life in high-income countries [1]. Both heavy and light motorcycles are used in Turkey. A recent study revealed that heavy motorcycles tend to be used in summer and light motorcycles are used mostly during spring and fall. Light motorcycles are generally used for work and transport, whereas heavy motorcycles are preferred for vacation and travel [2]. Different rider characteristics are related to the various types of motorcycles and motorcycle-related accidents. Motorcyclists are a vulnerable group of riders and have a particularly high accident risk [2]. Thus, there is a need for research to identify risks for the motorcyclist during a crash. The aims of this study were to identify subgroups of motorcyclists with a higher accident risk and evaluate the efficiency of protective clothing for preventing injuries [1].

## 2. Methods

A 1-year prospective study of motorcycle crashes was conducted beginning in June 2012. The participants in the study were patients involved in motorcycle crashes and admitted to our emergency department (ED). Participants were interviewed in the ED after their initial management. Information about the accident, motorcycle, helmet use, motorcycle clothing, injury location, speed, license, and alcohol consumption was recorded. The locations of the injuries were the head, face, thorax, abdomen, pelvis, spine, hands, forearm, arm, leg, feet, and soft tissue (abrasion and lacerations). Light motorcycles were considered to have an engine limit of 125 cm^3^, whereas heavy motorcycles were defined as those with an engine volume of >125 cc^3^ [2]. Motorcycle clothing was considered to include a helmet, jacket, pants, shoes, and gloves. Other equipment (boots, sport shoes, and coats) was not considered protective motorcycle clothing. This study obtained ethical approval from the Human Research Ethics Committee of our hospital.

First- and second-degree relatives were interviewed if the patient was unable to complete an informed consent form due to his or her medical condition. Patients were excluded from the study if they did not want to participate or a relative of the patient refused to provide informed consent.

Continuous data are presented as means and standard deviations, and categorical variables are presented as percentages. Yates' continuity correction, Fisher's exact test, Pearson's chi-square test, and odds ratios were used to evaluate the relationships among motorcycle type, protective clothing, and rider characteristics. *P* < 0.05 was considered significant, with a 95% confidence interval. Data were analyzed using NCSS 2007 and PASS 2008 statistical software.

## 3. Results

We identified 244 motorcycle crashes involving patients admitted to the ED during the study period. Eighteen patients did not provide informed consent due to their medical condition or declined to participate in the study and were excluded. Thus, 226 patients were included in the study. A total of 174 patients were involved in crashes with light motorcycles and 52 were involved in crashes with heavy motorcycles. 

Helmets and motorcycle pants were used by a significantly higher proportion of heavy than of light motorcycle riders (*P* < 0.05). Jackets, gloves, and shoes were more preferred by heavy than by light motorcycle riders (*P* < 0.01). No relationships were found among age, alcohol consumption, disability, mortality, seasonal distribution, or motorcycle type (*P* > 0.05, Table 1). In total, 68.3% of patients involved in light motorcycle crashes had a driver's license. This ratio was significantly lower than that for heavy motorcycle riders (*P* < 0.05, Table 1).

Patients involved in motorcycle accidents without helmets had a higher incidence of head and maxillofacial trauma compared with those who wore helmets (Table 2). Motorcycle jackets were not protective against thoracic, spinal, abdominal, or pelvic injuries or upper extremity fractures (*P* > 0.05). Patients who were not wearing motorcycle jackets when they were involved in motorcycle accidents had a higher incidence of upper body soft-tissue trauma compared with those who wore jackets (Table 2). Gloves were not protective against hand and wrist fractures (Table 2). Patients involved in motorcycle accidents without motorcycle gloves had a higher incidence of hand soft-tissue injuries compared with those who wore gloves (Table 2).

Motorcycle pants were not protective against leg and foot fractures. Patients involved in motorcycle accidents without motorcycle pants had a higher incidence of lower limb soft-tissue trauma compared with those wearing motorcycle pants (Table 2). Protective shoes were not protective against foot bone fractures but were protective against foot soft-tissue injuries (Table 2).

Patients involved in an accident with a light motorcycle were more likely to ride without a helmet than were heavy motorcycle riders (Table 3). Patients who rode without a driver's license had a higher incidence of riding without a helmet compared with those who had a license (Table 3), and those who consumed alcohol had a higher incidence of riding without a helmet than their nondrinking counterparts (Table 3). Patients involved in an accident with a light motorcycle had a higher incidence of riding without a jacket, special pants, shoes, and gloves than heavy motorcycle riders (Table 3), and those without a driver's license had a higher incidence of riding without a jacket, special pants, shoes, and gloves than those with a license (Table 3). 

## 4. Discussion

Kadıköy is one of the most culturally and economically developed parts of Turkey, and as such, it reveals unique features of motorcycle-related trauma epidemiology [3]. Due to the common use of heavy motorcycles and protective clothing, our hospital is a good place to conduct an evaluation of the effects of motorcycle type and protective clothing on injury patterns [3]. Previous studies have attempted to evaluate the epidemiological features of motorcycle-related injuries in Turkey. These studies were commonly conducted in rural parts of the country, were retrospective, and involved a very small number of patients [4–8]. There is a need for research to identify the risks during a motorcycle crash in Turkey.

Heavy motorcycles are preferred for vacations and travel [2], whereas light motorcycles are generally used for work and transport. Accidents with light motorcycles (76.9%) were more common in our study. The common use of light motorcycles is remarkable [1, 2, 9–11], but no associations were found between motorcycle type and rider age, seasonal distribution of accidents, alcohol consumption before the accident, morbidity, or mortality.

Drivers of light motorcycles were the most vulnerable riders. Helmets, motorcycle pants, jackets, gloves, and shoes were used significantly more often by heavy than by light motorcycle riders (*P* < 0.05). The proportion of riders with a license was significantly lower for light than for heavy motorcycle riders (*P* < 0.05). It is obvious that light motorcycles were more commonly involved in motorcycle crashes. Light motorcycle drivers did not prefer motorcycle protective clothing, and a significant proportion of them do not have sufficient training. Strict license controls and adding motorcycle protective clothing to motorcycle sales may reduce the incidence of injuries from light motorcycle crashes.

Patients involved in motorcycle accidents who were not wearing helmets had a higher incidence of head and maxillofacial trauma [12]. As traumatic brain injuries represent a significant cause of death and disability in young populations, helmets may decrease morbidity and mortality in light motorcycle riders, who were more likely to be involved in accidents in which they were not wearing a helmet [13, 14]. Riding without a motorcycle license and riding after consuming alcohol were other risk factors associated with riding without a helmet [9]. These findings are striking, as helmets have been shown to be effective [15–17].

Motorcycle jackets had no significant effect on systemic injuries but were effective against soft-tissue injuries. Motorcycle pants, shoes, and gloves were not protective for upper and lower extremity fractures, but they were protective against soft-tissue injuries [2].

Weather and driver age were not associated with wearing protective clothes [2, 10, 14]. However, riding without protective clothing was associated with driving without a license, use of a light motorcycle, and alcohol consumption [14]. 

Light motorcycle riders need to be strictly controlled. Alcohol consumption and riding without a license must be prevented [2, 15, 16]. Light motorcycle riders are the most vulnerable and the largest proportion of motorcyclists. They attract the majority of concern, as they are not trained or appear to be less serious about the risks of riding. 

Our results provide strong evidence for the benefits of motorcycle protective clothing in reducing soft-tissue injuries. However, there is a need for protective clothing to evolve to protect against fractures and systemic injuries.

Our study had limitations. Some patients may not have sought help after being involved in an accident, and patients who died during an accident might not be in our records. Protective clothing is not standardized due to the lack of global standards. 

## 5. Conclusion

Heavy motorcycles riders were safer than light motorcycle riders. Light motorcycle riders are the most vulnerable; they constitute the largest portion of motorcyclists and attract the majority of concern. Helmets may be effective for preventing head and facial injuries. Other protective clothes were not effective against fractures or systemic injuries but were effective against soft-tissue injuries. 

## Figures and Tables

**Table 1:** Key features of patients and use of protective clothing.

	Light (*n* = 174)	Heavy (*n* = 52)	*P*
Age < 30	63 (36,2%)	24 (46,2%)	0,258
Helmet	106 (60,9%)	40 (76,9%)	0,048
Jacket	55 (31,6%)	30 (57,7%)	0,001
Pants	46 (26,4%)	24 (46,2%)	0,011
Glove	49 (28,2%)	27 (51,9%)	0,001
Shoe	24 (13,8%)	21 (40,4%)	0,001
Alcohol	7 (4,0%)	2 (3,8%)	0,99
License	119 (68,3%)	47 (90,4%)	0,032
Dead	2 (1,1%)	1 (1,9%)	0,545
Disability	4 (2,3%)	2 (3,8%)	0,623
Summer	68 (39,1%)	26 (50,0%)	0,214
Winter	33 (19,0%)	5 (9,6%)	0,17
Fall	42 (24,1%)	6 (11,5%)	0,079
Spring	31 (17,8%)	15 (28,8%)	0,124

**Table 2:** Effects of protective wearing on injury.

	Helmet (+)	Helmet (−)	*P*	ODDS ratio (95% CI)
Head (brain injury)	2 (1,4%)	6 (7,5%)	0,025	5,838 (1,150–29,637)
Face traumas	20 (13,7%)	31 (38,8%)	0,001	3,986 (2,077–7,649)

	Jacket (+)	Jacket (−)		

Thoracic fractures	5 (5,9%)	5 (3,5%)	0,508	0,588 (0,165–2,095)
Spinal fractures	9 (10,6%)	5 (3,5%)	0,065	0,310 (0,100–0,960)
Abdominal injuries	3 (3,5%)	7 (50,0%)	0,747	1,428 (0,359–5,676)
Pelvic fractures	5 (5,9%)	6 (4,3%)	0,751	0,716 (0,212–2,423)
Upper extremity fractures	8 (9,4%)	13 (9,2%)	0,99	0,978 (0,388–2,465)
Upper body soft tissue	42 (49,4%)	112 (79,4%)	0,001	3,954 (2,193–7,130)

	Gloves (+)	Gloves (−)		

Hand/wrist fractures	28 (36,8%)	51 (34,0%)	0,672	0,883 (0,497–1,570)
Hand soft tissue	34 (44,7%)	120 (80,0%)	0,001	4,941 (2,702–9,0337)

	Pants (+)	Pants (−)		

Lower limb fractures	28 (40,0%)	60 (38,5%)	0,826	0,938 (0,527–1,669)
Lower limb soft tissue	30 (42,9%)	124 (79,5%)	0,001	5,167 (2,800–9,532)

	Shoes (+)	Shoes (−)		

Lower limb fractures	18 (40,0%)	70 (38,7%)	0,99	0,946 (0,485–1,843)
Lower limb soft tissue	18 (40,0%)	136 (75,1%)	0,001	4,533 (2,285–8,994)

**Table 3:** Factors affecting protective clothes wearing (^1^continuity correction (Yates) test, ^2^Pearson's chi-square test, ^3^Fisher's exact test, *P < 0.05, and **P < 0.01).

	Age > 30	Summer	License (−)	Alcohol (+)	Light motors
Helmet +	84 (57.5%)	68 (46.6%)	21 (14.4%)	2 (1.4%)	106 (72.6%)
Helmet −	55 (68.8%)	26 (32.5%)	39 (48.8%)	7 (8.8%)	68 (85.0%)
*P*	^ 2^ **0.098**	^2^0.040*	^1^0.001**	^3^0.011*	^1^0.048*
ODDS	1.624	0.552	5.662	6.904	2.138
95% CI	0.913–2.887	0.312–0.976	2.994–10.706	1.399–34.077	1.048–4.364

Jacket +	47 (55.3%)	36 (42.4%)	4 (4.7%)	2 (2.4%)	50 (64.7%)
Jacket −	92 (65.2%)	58 (41.1%)	56 (39.7%)	7 (5.0%)	119 (84.4%)
*P*	^ 2^ **0.136**	^ 2^ **0.857**	^1^0.001**	^ 3^ **0.489**	^1^0.001**
ODDS	1.518	0.951	13.341	2.168	2.950
95% CI	0.875–2.632	0.551–1.641	4.627–38.471	0.440–10.686	1.562–5.574

Pants +	38 (34.3%)	26 (37.1%)	2 (2.9%)	2 (2.9%)	46 (65.7%)
Pants −	101 (64.7%)	68 (43.6%)	58 (37.2%)	7 (4.5%)	128 (82.1%)
*P*	^ 2^ **0.135**	^ 2^ **0.827**	^1^0.001**	^ 3^ **0.724**	^1^0.011*
ODDS	1.546	1.308	20.122	1.597	2.835
95% CI	0.871–2.744	0.733–2.333	4.753–85.198	0.323–7.891	1.256–4.527

Glove +	42 (55.3%)	29 (38.2%)	4 (5.3%)	2 (2.6%)	49 (64.5%)
Glove −	97 (64.7%)	65 (43.3%)	56 (37.3%)	7 (4.7%)	125 (83.3%)
*P*	^ 2^ **0.170**	^ 2^ **0.456**	^1^0.001**	^ 3^ **0.721**	^2^0.001**
ODDS	1.482	1.239	10.723	1.811	2.755
95% CI	0.844–2.601	0.705–2.179	3.716–30.946	0.367–8.938	1.458–5.206

Shoe +	26 (57.8%)	17 (37.8%)	2 (4.4%)	1 (2.2%)	24 (53.3%)
Shoe −	113 (62.4%)	77 (42.5%)	58 (32.0%)	8 (4.4%)	150 (82.9%)
*P*	^ 1^ **0.687**	^ 1^ **0.681**	^1^0.001**	^ 3^ **0.692**	^1^0.001**
ODDS	1.214	1.219	10.138	2.035	4.234
95% CI	0.625–2.358	0.623–2.385	2.374–43.925	0.248–16.699	2.099–8.541

## References

[1] de Rome L, Ivers R, Fitzharris M (2011). Motorcycle protective clothing: protection from injury or just the weather?. *Accident Analysis and Prevention*.

[2] Bjornskau T, Navestad TO, Akhtar J (2012). Traffic safety among motorcyclists in Norway: a study subgroups and risk factors. *Accident Analysis & Prevention*.

[3] Erdoğan MO, Demir SA, Kosargelir M, Colak S, Öztürk E (2013). Local differences in the epidemiology of traumatic spinal injuries. *Turkish Journal of Trauma and Emergency Surgery*.

[4] Özkan S, İkizceli İ, Akdur O, Durukan P, Güzel M, Vardar A (2009). Injuries due to motorcycle accidents. *Journal of Academic Emergency Medicine*.

[5] Çetinus E, Ekerbiçer H (2000). Analysis of the motorcycle accidents in Kırıkhan, Antakya. *Turkish Journal of Trauma and Emergency Surgery*.

[6] Emet M, Beyhun NE, Uzkeser M, Aslan Ş (2008). Descriptive epidemiology of secondary transport from the emergency unit of a state hospital. *Turkish Journal of Medical Sciences*.

[7] Koçak S, Uçar K, Bayır A, Ertekin B (2010). Characteristics of the cases of bicycle and motorcycle accidents referred to the Emergency department. *Turkish Journal of Emergency Medicine*.

[8] Alıcıoğlu B, Yalnız E, Eskın D, Yılmaz B (2008). Injuries associated with motorcycle accidents. *Acta Orthopaedica et Traumatologica Turcica Journal*.

[9] De Rome L, Ivers R, Haworth N, Heritier S, Du W, Fitzharris M (2011). Novice riders and the predictors of riding without motorcycle protective clothing. *Accident Analysis and Prevention*.

[10] Ali M, Saeed MMS, Ali MM, Haidar N (2011). Determinants of helmet use behaviour among employed motorcycle riders in Yazd, Iran based on theory of planned behaviour. *Injury*.

[11] Teoh ER, Campbell M (2010). Role of motorcycle type in fatal motorcycle crashes. *Journal of Safety Research*.

[12] Ramli R, Abdul Rahman R, Abdul Rahman N (2008). Pattern of maxillofacial injuries in motorcyclists in Malaysia. *Journal of Craniofacial Surgery*.

[13] Söğüt Ö, Kaya H, Gökdemir MT (2012). Early oxidative status in adult patients with isolated traumatic brain injury. *Turkish Journal of Medical Sciences*.

[14] Schermer CR, Omi EC, Ton-That H (2008). A clustering of injury behaviors. *Journal of Trauma*.

[15] O’Keeffe T, Dearwater SR, Gentilello LM, Cohen TM, Wilkinson JD, McKenney MM (2007). Increased fatalities after motorcycle helmet law repeal: is it all because of lack of helmets?. *Journal of Trauma*.

[16] Liu BC, Ivers R, Norton R, Boufous S, Blows S, Lo SK (2008). Helmets for preventing injury in motorcycle riders. *Cochrane Database of Systematic Peviews*.

[17] Grange JT, Cotton A (2004). Motorsports medicine. *Current Sports Medicine Reports*.

